# HIF and COX-2 expression in triple negative breast cancer cells with hypoxia and 5-fluorouracil

**Published:** 2020-11-01

**Authors:** Noriko Mori, Yelena Mironchik, Flonné Wildes, Sherry Y. Wu, Kanami Mori, Balaji Krishnamachary, Zaver M. Bhujwalla

**Affiliations:** 1 Division of Cancer Imaging Research, Russell H. Morgan Department of Radiology and Radiological Science, Johns Hopkins University School of Medicine, Baltimore, Maryland 21205, USA; 2 Sidney Kimmel Comprehensive Cancer Center, Johns Hopkins University School of Medicine, Baltimore, Maryland 21231, USA; 3 Department of Radiation Oncology and Molecular Radiation Sciences, Johns Hopkins University School of Medicine, Baltimore, Maryland 21205, USA

**Keywords:** 5-FU, COX-2, HIF, Hypoxia, TNBC

## Abstract

Our purpose was to understand the effects of normoxia or hypoxia on 5-fluorouracil (5-FU) treatment in triple negative breast cancer (TNBC) cells, and characterize the molecular changes in hypoxia inducible factors (HIFs) and cyclooxygenase-2 (COX-2) following treatment. Cell viability and protein levels of HIFs and COX-2 were determined after wild type and HIF silenced MDA-MB-231 cells, and wild type SUM-149 cells, were treated with 5-FU under normoxia or hypoxia. 5-FU reduced cell viability to the same levels irrespective of normoxia or hypoxia. HIF silenced MDA-MB-231 cells showed comparable changes in cell viability, supporting observations that hypoxia and the HIF pathways did not significantly influence cell viability reduction by 5-FU. Our data suggest that HIF-2*α* accumulation may predispose cancer cells to cell death under hypoxia. SUM-149 cells that have higher COX-2 and HIF-2*α* following 24 h of hypoxia, were more sensitive to 96 h of hypoxia compared to MDA-MB-231 cells, and were more sensitive to 5-FU than MDA-MB-231 cells. COX-2 levels changed with hypoxia and with 5-FU treatment but patterns were different between the two cell lines. At 96 h, COX-2 increased in both untreated and 5-FU treated cells under hypoxia in MDA-MB-231 cells. In SUM-149 cells, only treatment with 5-FU increased COX-2 at 96 h of hypoxia. Cells that survive hypoxia and 5-FU treatment may exhibit a more aggressive phenotype. Our results support understanding interactions between HIF and COX-2 with chemotherapeutic agents under normoxia and hypoxia, and investigating the use of COX-2 inhibitors in these settings.

## 1 Introduction

Although surgery is the primary form of treatment for breast cancer, some form of systemic neoadjuvant therapy is increasingly given prior to surgery^[1]^. Understanding the impact of these systemic treatments and the molecular changes induced by these treatments, especially within the abnormal microenvironments of tumors, therefore becomes important within the context of relapse. 10–30 % of all breast cancer cases are triple-negative breast cancer (TNBC)^[2]^. TNBCs, so defined because of the lack of expression of estrogen receptor (ER), progesterone receptor, and human epidermal growth factor receptor 2 (HER2)^[3]^, typically have early relapse and poor overall survival^[4]^. Identifying mechanisms that may contribute to relapse is of significant importance in developing treatment strategies to overcome the poor prognosis of TNBC.

The abnormal vasculature that exists in tumors results in poor drug delivery as well as in the formation of hypoxic areas^[5]^. Hypoxia results in the stabilization of hypoxia inducible factors (HIFs) that activate the transcription of numerous genes involved in angiogenesis, metabolism, invasion, metastasis, the immune system, and resistance to chemo- and radiation therapy^[6–8]^. There are three isoforms of the *α* subunit, HIF-1*α*, HIF-2*α*, and HIF-3*α*. HIF-1*α* is frequently elevated in TNBC^[9]^. HIF-2*α* has been shown to correlate to distant recurrence and poor outcome in breast cancer^[10]^. Both play a major role in breast cancer cell metabolism^[11]^, although they have been found to have opposing roles in hypoxic tumor growth^[12]^ and metabolism^[13]^. Both HIF-1*α* and HIF-2*α* are important in the metastatic cascade^[11]^. Cyclooxygenase-2 (COX-2), a rate-limiting enzyme in prostaglandin synthesis, is overexpressed in various cancers, including breast cancers^[14,15]^. Hypoxia has been found to induce COX-2 expression in various cells and tissues including pulmonary artery smooth muscle, epithelial cells, and colorectal tumors^[16,17]^. HIF-1*α* or HIF-2*α* has been found to regulate COX-2 expression^[16–20]^. Inhibition of COX-2 was found to reduce metastasis formation in breast cancer cells and xenografts^[21–23]^, and in women with breast cancer^[24]^.

Because of the abnormal tumor vasculature and poor drug delivery, cancer cells *in vivo* are exposed to a range of drug concentrations under normoxic and hypoxic conditions. These drugs are metabolized or cleared through perfusion. The damage to these cells, as well as the molecular changes induced by the drug under these conditions, evolves over a period of time. We therefore treated two TNBC cell lines, MDA-MB-231 and SUM-149, and three sublines of MDA-MB-231 cells engineered to stably express shRNA against HIF-1*α*, HIF-2*α* and both HIF-1*α* and HIF-2*α* with varying doses of 5-FU. HIF-1*α*, HIF-2*α* and COX-2 levels were characterized at multiple time points after treatment with 5-FU. 5-FU, a synthetic fluorinated pyrimidine analogue that inhibits RNA synthesis^[25,26]^, or its prodrug capecitabine, form an integral part of neoadjuvant therapy of TNBC^[27]^. Cells were maintained under normoxia or hypoxia during 5-FU treatment, as well as during multiple time points after treatment, to mimic conditions *in vivo*.

## 2 Materials and methods

### 2.1 Cell culture and treatment

Triple negative MDA-MB-231 human breast cancer cells were obtained from American Type Culture Collection (ATCC, Manassas, VA), and grown in RPMI-1640 medium supplemented with 10 % fetal bovine serum (FBS; Sigma-Aldrich, St. Louis, MO). Triple negative SUM-149 inflammatory human breast cancer cells were obtained from Asterand (Detroit, MI), and were grown in DMEM/F12 (1:1) medium (Mediatech, Manassas, VA) supplemented with 5% FBS, 5 *μ*g/ml insulin (Life Technologies, Grand island, NY) and 0.5 *μ*g/ml hydrocortisone (Sigma-Aldrich). MDA-MB-231 cells stably expressing shRNA against HIF-1*α* (231-sh-HIF-1*α*), HIF-2*α* (231-sh-HIF-2*α*) and both HIF-1*α* and HIF-2*α* (231sh-HIF-1/2*α*) were generated using lentiviral transduction as previously described^[11,28]^. These genetically engineered MDA-MB-231 sublines were maintained in RPMI-1640 medium supplemented with 10 % fetal bovine serum. All cell lines were maintained in a humidified atmosphere with 5 % CO_2_ in air at 37°C, and were tested routinely for mycoplasma contamination. Hypoxic treatment of cells was performed by placing the plates or dishes in a modular incubator chamber (Billups-Rothenberg, Del Mar, CA) flushed at 2 p.s.i. for 3 minutes with a gas mixture of 1% O_2_, 5% CO_2_ and N_2_ for balance.

Cells were treated with 0.5 to 50 *μ*g/ml 5-FU (Fresenius Kabi USA, LLC, Lake Zurich, IL) or Hanks’ balanced salt solution (HBSS, Sigma-Aldrich) as control for 24 h under normoxia (N, 20% O_2_) or hypoxia (H, 1% O_2_). After 24 h treatment (N 24 h or H 24 h), cells were analyzed for proliferation or protein expression. In additional batches of cells, medium with 5-FU or HBSS was changed to fresh culture medium without 5-FU or HBSS under normoxia within 15 minutes and cells were continued to be cultured under normoxia or hypoxia for another 24 h (N 48 h or H 48 h), 48 h (N 72 h or H 72 h), or 72 h (N 96 h or H 96 h) after 5-FU treatment. Cells were analyzed for proliferation or protein expression at these time points.

### 2.2 Cell viability/proliferation assay by CCK-8

5000 cells were seeded in each well of a 96 well plate and cultured overnight. Twenty-four hours later, cells were treated with 5-FU for 24 h under normoxia or hypoxia. Cell viability was determined at various time points using cell counting kit-8 assays (CCK-8, Dojindo Molecular Technologies, Inc. MD), performed using the manufacturer’s instructions. Cell viability was measured at 450 nm using a 1420 Multilabel counter (Perkin Elmer, Waltham, MA) after 2 h incubation under normoxia with the CCK-8 reagent. In the CCK-8 colorimetric assay the amount of the formazan dye, which is generated by the activities of dehydrogenases in cells, is directly proportional to the number of living cells. Untreated cells and HBSS treated cells were used as negative controls. Values from each group were normalized to the average of values obtained from untreated normoxic cells that was set to 100% viability. We made sure that the incubation time (2 h) after adding the CCK-8 reagent and blank absorbance was similar in samples from N and H conditions to allow for comparisons. At least 3 independent experiments were performed.

### 2.3 Immunoblot analysis

Whole-cell extracts were prepared by lysing cells with RIPA lysis buffer supplemented with a protease inhibitor cocktail (Sigma-Aldrich, St Louis, MO, USA). Protein concentrations were estimated using the Bradford’s method (Bio-Rad, Hercules, CA). Equal amounts of total protein were resolved on a 7.5% SDS-PAGE gels and transferred to a nitrocellulose membrane (Bio-Rad). After blocking in 5% milk-TBST (TBS Tween), the membrane was separately probed with HIF-1*α* antibody (BD Biosciences San Jose, CA), HIF-2*α* antibody (Novus Biologicals, Littleton, CO) and COX-2 antibody (Cayman Chemical, Ann Arbor, MI). Anti-GAPDH antibody (Sigma-Aldrich) was used for equal loading assessment. Secondary antibodies were horseradish peroxidase conjugated anti-mouse, anti-rabbit (GE Healthcare, Chicago, IL), or anti-goat IgG (Novus Biologicals). The signal was visualized using ECL Plus reagents (Thermo Scientific, Rockford, IL) and developed on a Blue Bio film (Denville Scientific, Metuchen, NJ). The films were scanned and densitometric analysis was performed using the ImageJ (NIH, Bethesda, MD). Relative density changes against GAPDH were analyzed and in some cases normalized to the immunoreactive bands in control cells (HBSS treated), which was set to 1 using data from 2–3 cell lysates.

### 2.4 Statistical analysis

Data are expressed as Mean ± Standard Error Mean (SEM). Statistical significance was evaluated using a one-tailed unpaired Student’s t-test. P values ≤ 0.05 were considered to be significant.

## 3 Results

### 3.1 Effects of 5-FU under normoxia or hypoxia on cell viability in MDA-MB-231 cells

**Figure 1.**
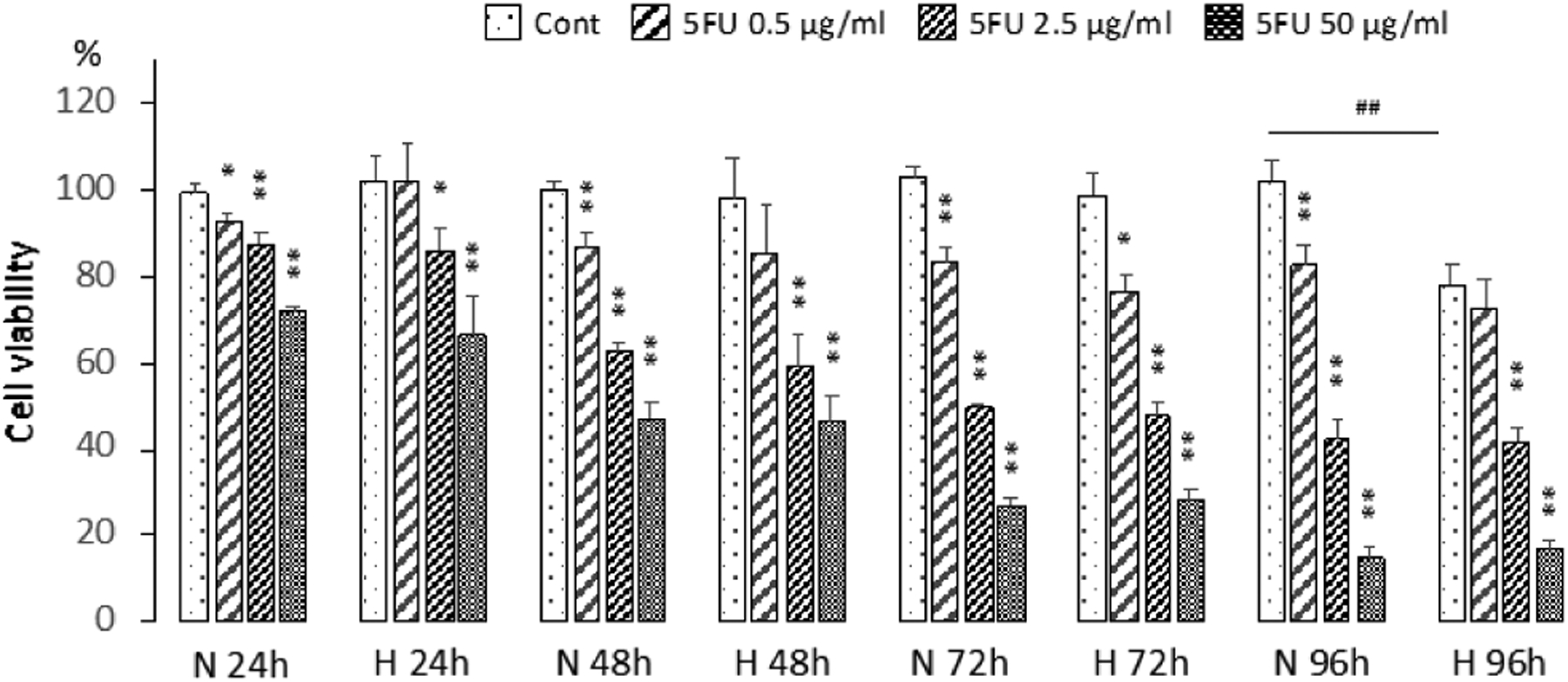
Cell viability (%) normalized to untreated cells under normoxia (100%) (n ≥ 3). MDA-MB-231 wild type (WT) cells were exposed to normoxia (N) or hypoxia (H) in the presence or absence of 0.5–50 *μ*g/ml 5-FU for 24 h (N or H 24 h), following which medium was changed to fresh growth medium, and cells were cultured for another 24 h (N or H 48h), 48 h (N or H 72 h), and 72 h (N or H 96 h) under normoxia or hypoxia after which CCK-8 assays were performed. Values from each group were normalized to the average of values obtained from untreated normoxic cells that was set to 100% viability. Cont: HBSS treated cells. Values represent Mean ± SEM. ** P ≤ 0.01, * P ≤ 0.05, between Cont and 5-FU treatment at the same time point. ^##^ P ≤ 0.01, between N and H.

Cell viabilities (%) of the different groups compared to untreated cells under normoxia are shown in Figure 1. The three doses of 0.5, 2.5, and 50 *μ*g/ml were selected to represent low toxicity (~80% cell viability at 96 h), medium toxicity (~40–50% cell viability at 96 h), and high toxicity (~15% cell viability at 96 h).

MDA-MB-231 cells, maintained under 96 h hypoxia, showed significantly lower cell viability (77.7%) compared to normoxic cells at the same time point. Treatment with the highest dose of 50 *μ*g/ml 5-FU resulted in a significant decrease of viability at all the time points with a greater reduction of cell viability with increasing time, even though 5-FU was withdrawn at 24 h. This dose was equally effective under normoxic or hypoxic conditions.

Similar to the higher dose, treatment with 0.5 *μ*g/ml and 2.5 *μ*g/ml 5-FU resulted in a progressive reduction of cell viability at the later times, although the decrease of cell viability was less than that for the higher dose. The lower doses were also equally effective under normoxic or hypoxic conditions in these cells.

### 3.2 Effects of 5-FU under normoxia or hypoxia on HIF-1*α*, HIF-2*α* and COX-2 in MDA-MB-231 cells

**Figure 2.**
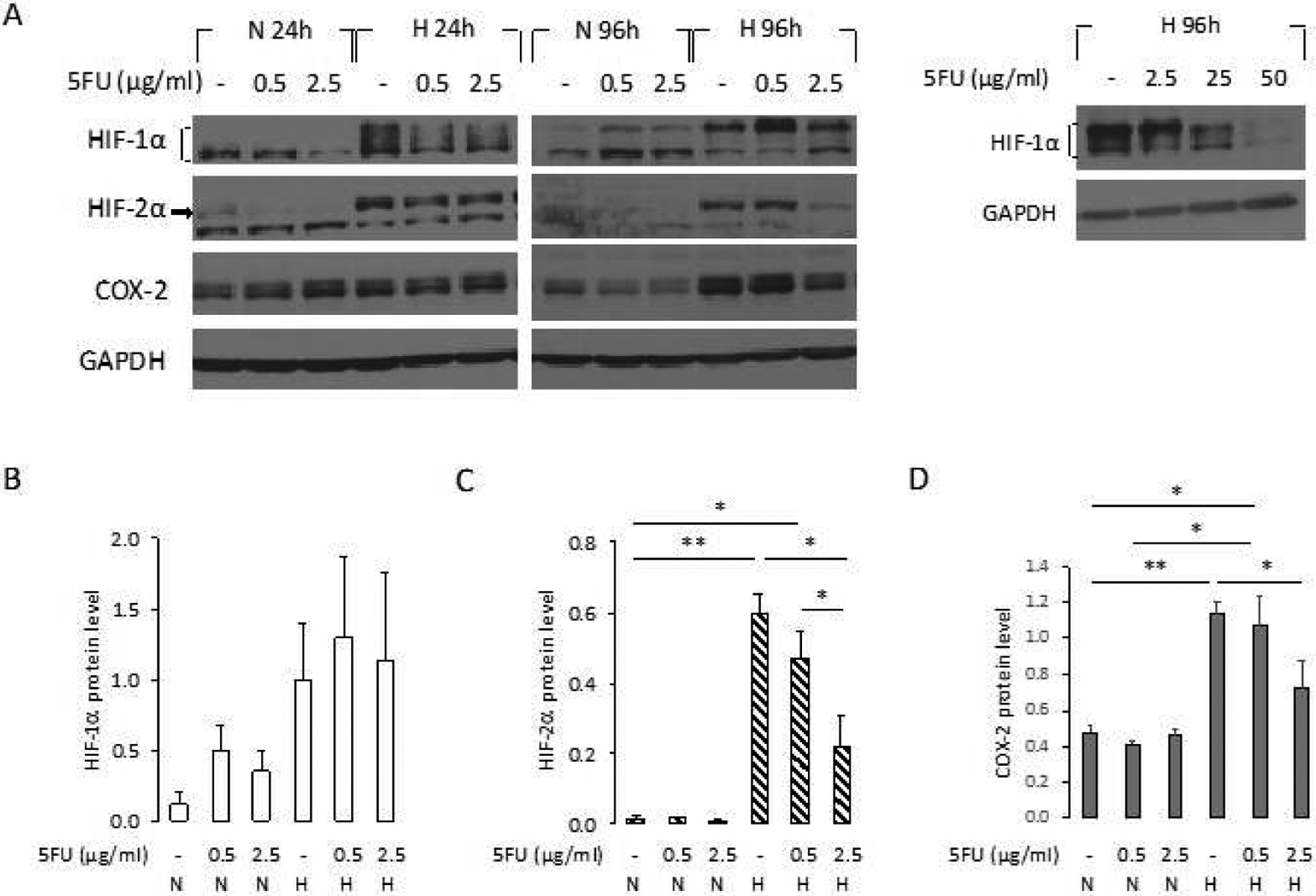
(A) Protein levels of HIF-1*α*, HIF-2*α* and COX-2 in MDA-MB-231 WT cells were determined by immunoblot analysis after treatment with HBSS (−) or 0.5 – 2.5 *μ*g/ml 5-FU for 24 h (N 24h, H 24h) or after medium was changed to fresh growth medium, and cells were cultured another 72h (N 96h, H 96h). Also shown (right panel) are HIF-1*α* protein levels by immunoblot analysis after treatment with HBSS or 2.5 – 50 *μ*g/ml 5-FU at H 96 h. GAPDH protein levels were used for equal loading assessment. Relative density changes in (B) HIF-1*α*, (C) HIF-2*α*, and (D) COX-2 protein levels, normalized to GAPDH protein levels, obtained using ImageJ at N 96 h (n = 3). Values represent Mean ± SEM. ** P < 0.01, * P ≤ 0.05.

To understand the molecular changes in these cells, we characterized HIF-1*α*, HIF-2*α*, and COX-2 protein levels under different conditions. Since cell viability was most reduced with 5-FU treatment at N or H 96 h, we compared protein levels of HIF-1*α*, HIF-2*α* and COX-2 at N and H 24 h and N and H 96 h for 5-FU doses of 0.5 *μ*g/ml and 2.5 *μ*g/ml. Representative immunoblots from these studies are presented in Figure 2A. As anticipated, hypoxia increased HIF-1*α* and HIF-2*α* expression at H 24 h compared to N 24 h irrespective of the presence of absence of 5-FU. At N or H 24 h, COX-2 levels remained unchanged from control levels (Figure 2A).

At H 96 h, HIF-1*α* level remained unchanged irrespective of the presence or absence of 5-FU up to doses of 2.5 *μ*g/ml (Figure 2A and 2B). However, at higher 5-FU doses of 25 and 50 *μ*g/ml, HIF-1*α* decreased at H 96 h (Figure 2A). HIF-2*α* and COX-2 also decreased at these higher doses of 5-FU (data not shown). Unlike HIF-1*α* that required much higher doses to decrease, at H 96 h, HIF-2*α* significantly decreased following treatment with 2.5 *μ*g/ml of 5-FU but not with the lower dose of 0.5 *μ*g/ml (Figure 2A and 2C). At H 96 h, COX-2 levels significantly increased in untreated cells or cells treated with 0.5 *μ*g/ml of 5-FU, suggesting that hypoxia drove up COX-2, but started to decrease at the 2.5 *μ*g/ml dose of 5-FU suggesting that like HIF-2*α*, COX-2 also decreased with 5-FU treatment. Changes of COX-2 at 96 h are summarized in Figure 2D.

### 3.3 Effects of 5-FU under normoxia or hypoxia on cell viability in HIF-1*α* and HIF-2*α* silenced MDA-MB-231 cells

**Figure 3.**
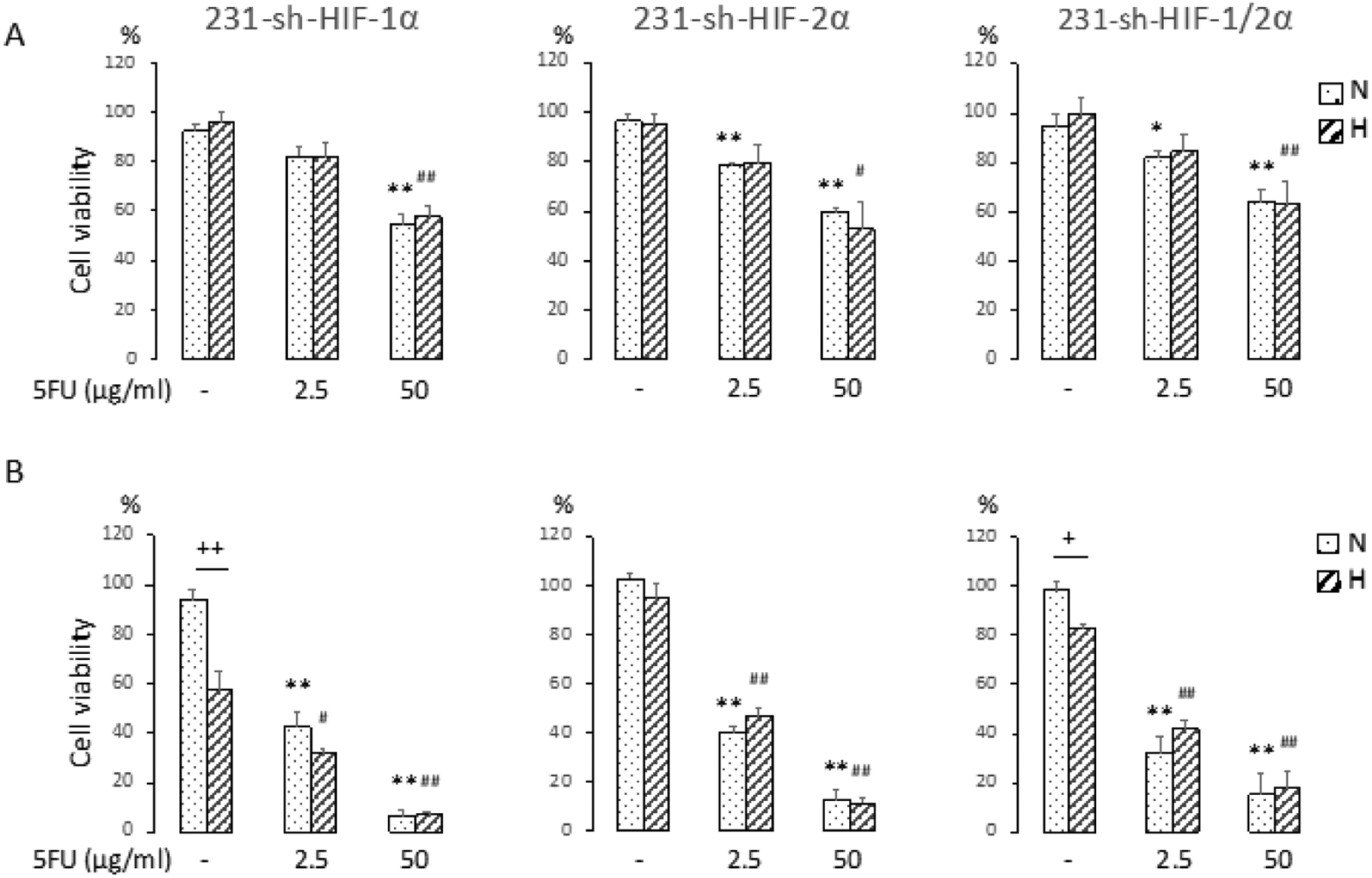
(A) Cell viability (%) at 24h and (B) Cell viability (%) at 96h (n ≥ 3). MDA-MB-231 sublines, 231-sh-HIF-1*α*, 231-sh-HIF-2*α*, 231-sh-HIF-1/2*α* cells were exposed to normoxia (N) or hypoxia (H) in the presence or absence of 2.5 or 50 *μ*g/ml 5-FU for 24 h. For N and H 96 h, medium was changed to fresh growth medium after 24h 5-FU treatment, cells were cultured another 72 h under N or H, and cell viability assays were performed. Values from each group were normalized to the average of values obtained from untreated normoxic cells that was set to 100% viability. Values represent Mean ± SEM. **, ^##^P < 0.01, ^#^ P ≤ 0.05, between Cont and 5-FU treatment in N or H. ^+^ P ≤ 0.05, between N and H.

To further validate the cell viability data, we characterized the effects of hypoxia on cell viability following 5-FU treatment using HIF-1*α*, or HIF-2*α*, or HIF-1*α* and HIF-2*α* silenced MDA-MB-231 cells. As in the studies with MDA-MB-231 wild type cells, MDA-MB-231 sublines were treated with 2.5 *μ*g/ml or 50 *μ*g/ml 5-FU under normoxia or hypoxia and cell viability were determined. As shown in Figure 3A, downregulating HIF-1*α*, or HIF-2*α*, or HIF-1*α* and HIF-2*α* resulted in comparable decreases of cell viability between normoxic and hypoxic conditions with 5-FU treatment at 24 h, providing further evidence that 5-FU was almost equally effective in reducing cell viability, irrespective of the presence or absence of hypoxia and the silencing of HIF pathways. The same effectiveness was observed at 96 h (Figure 3B). The changes in cell viability were also comparable to the wild type MDA-MB-231 cells (compare to Figure 1). Control cells from HIF-1*α* silenced cells, and HIF-1*α* and HIF 2*α* silenced cells, but not HIF-2*α* silenced cells showed a decrease of cell viability at 96 h under hypoxia. Since wild type cells also showed a decrease of cell viability at 96 h under hypoxia, these data suggest that silencing of HIF-2*α* alone seemed to improve cell survival under hypoxic conditions, but not against the treatment with 5-FU.

### 3.4 Effects of 5-FU under normoxia or hypoxia on HIF-1*α*, HIF-2*α* and COX-2 in HIF-1*α* and HIF-2*α* silenced MDA-MB-231 cells

**Figure 4.**
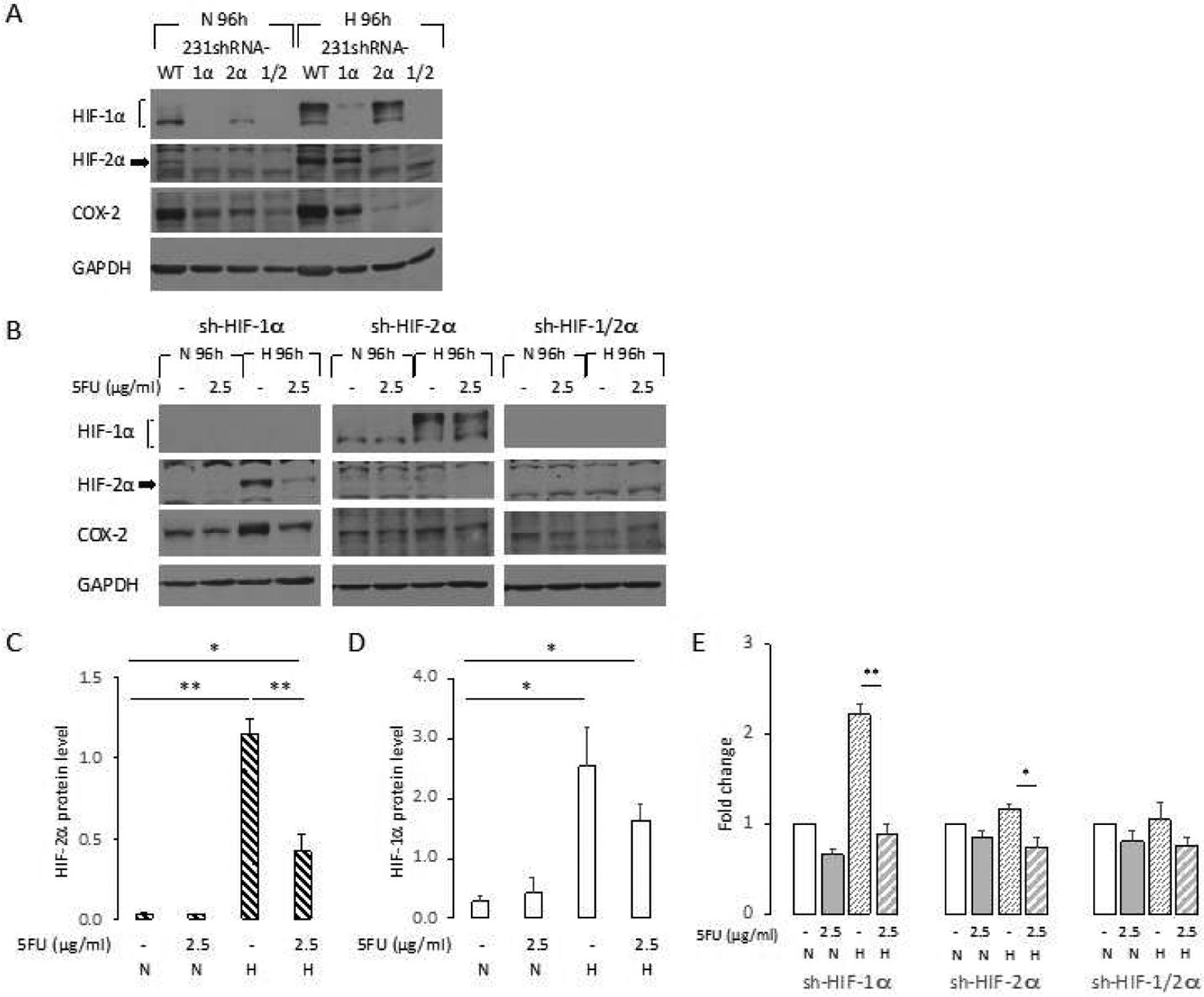
(A) Protein levels of HIF-1*α*, HIF-2*α* and COX-2 were determined by immunoblot analysis after culturing HBSS treated (control) MDA-MB-231 WT, 231-sh-HIF-1*α* (1*α*), 231-sh-HIF-2*α* (2*α*), and 231-sh-HIF-1/2*α* (1/2) cells for 96 h N or H. (B) Protein levels of HIF-1*α*, HIF-2*α* and COX-2 in 231-sh-HIF-1*α* (sh-HIF-1*α*), 231-sh-HIF-2*α* (sh-HIF-2*α*), and 231-sh-HIF-1/2*α* (sh-HIF-1/2*α*) cells were determined by immunoblot analysis after the treatment with HBSS (−) or 2.5 *μ*g/ml 5-FU at N and H 24h, and N and H 96h. Relative density changes in (C) HIF-2*α* in 231-sh-HIF-1*α* cells (n = 4), and (D) HIF-1*α* (n = 3) in 231-sh-HIF-2*α* cells, normalized to GAPDH protein levels, were obtained using ImageJ at N 96 h. (E) Relative density changes in COX-2 protein level against GAPDH protein level obtained using ImageJ, and normalized to the value obtained in control cells at N 96 h (n ≥ 3). Values represent Mean ± SEM. ** P < 0.01, * P ≤ 0.05.

Immunoblots of HIF-1*α*, HIF-2*α*, and COX-2 expression in wild type, HIF-1*α*, HIF-2*α*, and HIF1*α* and HIF-2*α* silenced control cells at 96 h normoxia or hypoxia are shown in Figure 4A. As anticipated there was almost non-detectable expression of the corresponding HIF silenced protein under normoxia or hypoxia compared to the wild type cells. The regulation of COX-2 expression by HIF is evident from the increase of COX-2 under hypoxic conditions in the wild type cells and the decrease of COX-2 in the HIF silenced cells under normoxia at 96h (Figure 4A). Immunoblots of HIF-1*α*, HIF-2*α*, and COX-2 expression in HIF-1*α*, HIF-2*α*, and double silenced cells at N 96 h and H 96 h with or without 2.5 *μ*g/ml 5-FU treatment are shown in Figure 4B. As with wild type cells (Figure 2A), under hypoxic conditions, HIF-2*α* significantly decreased in the HIF-1*α* silenced cells following treatment with 5-FU (Figure 4B and 4C). HIF-1*α* tended to decrease in HIF-2*α* silenced cells following treatment with 5-FU, but the changes were not significant (Figure 4B and 4D).

Like the wild type cells, COX-2 significantly increased with hypoxia in the HIF-1*α* silenced cells, with the increase eliminated in 5-FU treated cells (Figure 4E). Although the increase of COX-2 under hypoxia was much less pronounced in HIF-2*α* silenced cells, 5-FU treatment resulted in a decrease. In double silenced cells we did not observe an increase of COX-2 with hypoxia; COX-2 levels did not change with 5-FU treatment in these cells (Figure 4E).

### 3.5 Effects of 5-FU under normoxia or hypoxia on cell viability, HIF-1*α*, HIF-2*α* and COX-2 in SUM-149 cells

**Figure 5.**
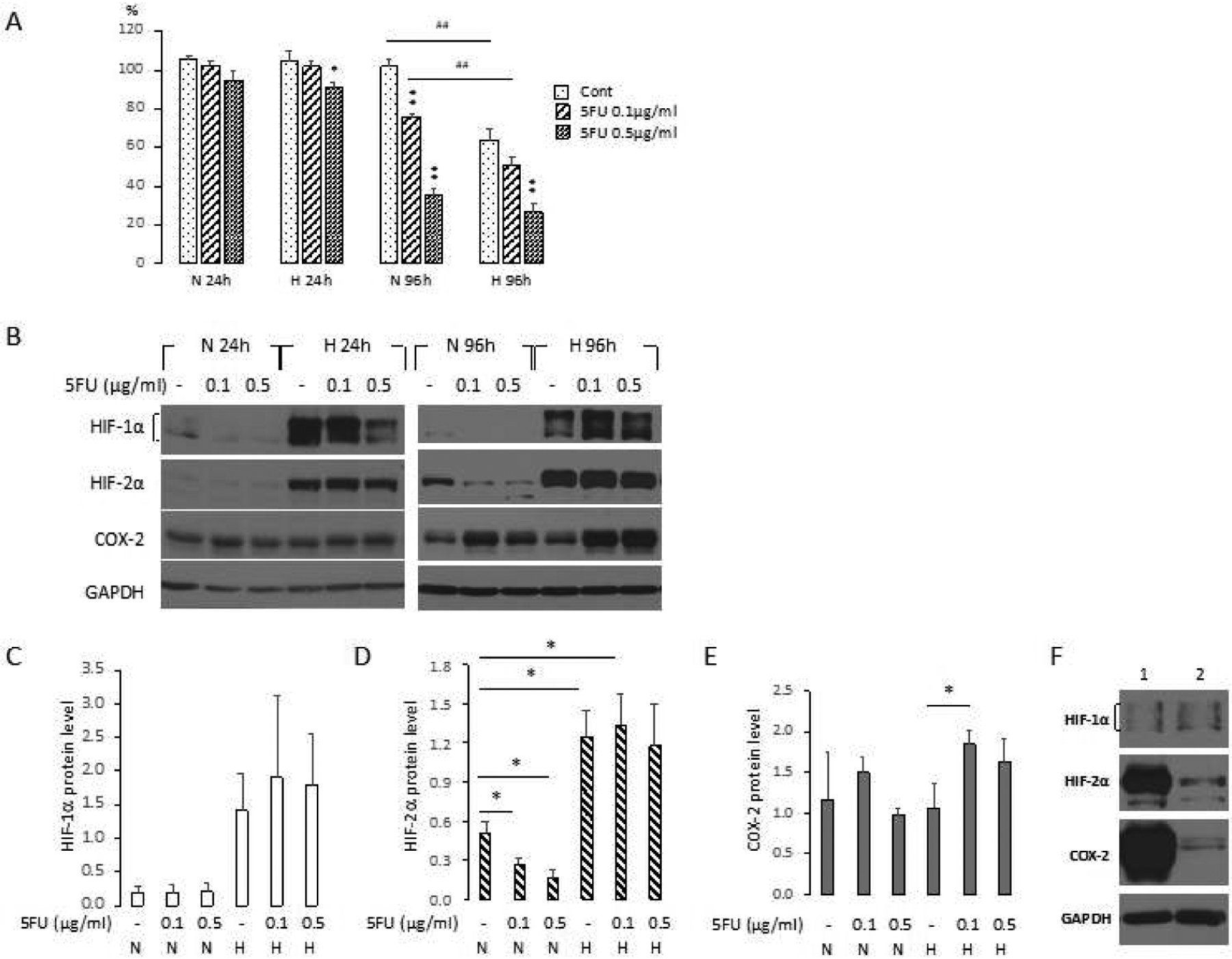
(A) Cell viability (%) compared to untreated cells under normoxia (100%) (n = 4). SUM-149 cells were exposed to normoxia (N) or hypoxia (H) in the presence or absence of 0.1 or 0.5 *μ*g/ml 5-FU for 24h (N or H 24 h), following which medium was changed to fresh growth medium under normoxia, and cells were cultured another 72 h (N or H 96 h) under normoxia or hypoxia after which CCK-8 assays were performed. Values from each group were normalized to the average of values obtained from untreated normoxic cells that was set to 100% viability. Cont: HBSS treated cells. Values represent Mean ± SEM. ** P < 0.01, * P ≤ 0.05, between Cont and 5-FU treatment at the same time point. ^##^ P < 0.01, between N and H. (B) Protein levels of HIF-1*α*, HIF-2*α* and COX-2 in SUM-149 cells were determined by immunoblot analysis after treatment with HBSS (−) or 0.1 – 0.5 *μ*g/ml 5-FU for 24 h (N 24h, H 24h) or after medium was changed to fresh growth medium, and cells were cultured another 72 h (N 96h, H 96h). Relative density changes in (C) HIF-1*α* (n = 2), (D) HIF-2*α* (n = 3), and (E) COX-2 protein levels (n = 3), normalized to GAPDH protein levels, obtained using ImageJ at N 96 h. control cells at N 96 h (n ≥ 3). Values represent Mean ± SEM. ** P < 0.01, * P ≤ 0.05. (F) Comparison of protein levels of HIF-1*α*, HIF-2*α* and COX-2 in (1) SUM-149 WT and (2) MDA-MB-231 WT at H 24 h.

We next used a second TNBC cell line, the inflammatory cell line SUM-149, to further characterize the role of HIF and COX-2 in the response of TNBC cells to 5-FU under normoxia and hypoxia. As shown in Figure 5A, these cells were significantly more sensitive to 5-FU compared to MDA-MB-231 cells, with a dose of 0.5 *μ*g/ml resulting in a reduction of cell viability comparable or even more effective than a dose of 2.5 *μ*g/ml in MDA-MB-231 cells. As shown in Figure 5A, hypoxia resulted in a significant decrease of cell viability in control cells and cells treated with 0.1 *μ*g/ml compared to normoxic cells, suggesting that hypoxia played a dominant role in the reduction of cell viability at this very low dose. However, at 0.5 *μ*g/ml there were no differences in viability between normoxic and hypoxic cells.

Immunoblot characterization of changes in HIF-1*α*, HIF-2*α* and COX-2 for N and H 24 h and 96 h are shown in Figure 5B and changes of HIF-1*α*, HIF-2*α* and COX-2 level for the 96 h time point are summarized in Figure 5C–5E. At H 24 h there was an increase of HIF-1*α* and HIF-2*α* but not COX-2. Treatment with 0.5 *μ*g/ml of 5-FU decreased induction of HIF-1*α* protein level at H 24 h (Figure 5B). The levels of HIF-2*α* and COX-2 proteins were not modified by 5-FU treatment at H 24 h. At H 96 h, HIF-1*α* increased slightly following treatment with 0.1 and 0.5 *μ*g/ml of 5-FU although this was not significant, whereas HIF-2*α* levels remained unchanged with treatment (Figure 5B, 5C and 5D). At N 96 h, HIF-2*α* levels decreased significantly following treatment with 0.1 and 0.5 *μ*g/ml of 5-FU (Figure 5B and 5D). At H 96 h COX-2 level significantly increased with 5-FU treatment (Figure 5E). The patterns observed with SUM-149 cells were very different from those observed with MDA-MB-231 cells. A comparison of HIF-1*α*, HIF-2*α* and COX-2 displayed in Figure 5F shows the much higher levels of HIF-2*α* and COX-2 in the SUM-149 cells compared to the MDA-MB-231 cells at H 24 h; baseline levels of COX-2 under normoxic conditions were similar to those observed under hypoxia in SUM-149 cells, but hardly detectable in MDA-MB-231 cells.

## 4 Discussion and conclusion

We found that 5-FU decreased cell viability to similar levels irrespective of cells being maintained under normoxia or hypoxia (24 h to 96 h), in MDA-MB-231 and SUM-149 triple negative human breast cancer cells. This was further validated using HIF silenced MDA-MB-231 cells that showed comparable changes in cell viability supporting the observation that hypoxia and the HIF pathways did not significantly influence cell viability reduction by 5-FU at the doses used, at least in culture. Previous studies investigating the effects of hypoxia on sensitivity to 5-FU have shown a cell dependent effect^[29]^. Anemia in patients has been associated with relapse in breast cancer patients receiving cyclophosphamide/methotrexate/5-FU chemotherapy^[30]^. In general, 5-FU is thought to be less effective under hypoxia^[31,32]^. At 0.1% hypoxia, MDA-MB-231 cells showed an almost twofold increase of IC_50_ (14 *μ*M *vs* 39 *μ*M)^[33]^. In our studies with MDA-MB-231 and SUM-149 cells we found no differences in cell viability with 1% hypoxia following 24 h 5-FU treatment at doses of 0.5 *μ*g/ml (3.8 *μ*M) to 50 *μ*g/ml (384 *μ*M) that was additionally confirmed in HIF silenced cells. Our data suggest that the reduction of cell viability following 5-FU treatment was not dependent upon HIF regulated pathways in these two TNBC cell lines.

Consistent with its known role in mediating the adaptive response of cells to hypoxia^[12]^, silencing HIF-1*α* resulted in a greater reduction of cell viability following 96 h of hypoxia. Interestingly, HIF-2*α* silencing improved MDA-MB-231 cell survival under hypoxic conditions, suggesting that HIF-2*α* accumulation may predispose these cancer cells to cell death under hypoxia. Because of these opposing effects, silencing both HIF-1*α* and HIF-2*α* resulted in lesser reduction of cell viability than silencing HIF-1*α* alone. Our observations with HIF-2*α* are consistent with previous observations in neuroblastoma where HIF-2*α* was observed to be a key component in tumor response to azacytidine combined with retinoic acid, and a small molecule inhibitor of HIF-2*α* reduced tumor response^[34]^. Similarly, epigenetic reexpression of HIF-2*α* was found to suppress soft tissue sarcoma growth[35].

SUM-149 cells that were characterized by high basal COX-2 levels and significantly higher induction of HIF-2*α* following 24 h of hypoxia, compared to MDA-MB-231 cells, were more sensitive to a reduction of cell viability with 96 h of hypoxia (64%) compared to MDA-MB-231 cells (78%), further supporting the role of HIF-2*α* in predisposing cancer cells to death. SUM-149 cells were also more sensitive to 5-FU than MDA-MB-231 cells with a dose of 0.5 *μ*g/ml resulting in a reduction of cell viability that was comparable or even more effective than a dose of 2.5 *μ*g/ml in MDA-MB-231 cells.

The patterns of changes in HIF and COX-2 with hypoxia and with 5-FU treatment were different between the two cell lines. At 24 h, treatment with 5-FU did not induce COX-2 in either of the cell lines irrespective of normoxia or hypoxia. In MDA-MB-231 cells at 96 h, COX-2 increased in both control and in 5-FU treated cells under hypoxia but not under normoxia. This increase of COX-2 in control cells was also pronounced in HIF-1*α* silenced cells but was diminished in HIF-2*α* silenced cells, and eliminated in combined HIF-1 and HIF-2*α* silenced cells, suggesting that the increase of COX-2 under hypoxia may be mediated through HIF-2*α*. HIF-2*α* has been previously observed to regulate the COX2/mPGES-1/PGE2 pathway in colon cancer^[18]^. On the other hand, in SUM-149 cells at 96 h, COX-2 increased only with combined hypoxia and 5-FU treatment, but not with hypoxia alone despite a robust increase of HIF-2*α* in the control cells. Factors such as cell-cell contact and acidic medium conditions that also exist in tumors may have also contributed to these differences.

In a study of biopsy samples from 14 esophageal carcinomas, COX-2 expression increased following 5-FU chemotherapy^[36]^. In the same study, cancer cells obtained from a panel of tumors including breast tumors exposed to 170 *μ*M 5-FU for 6 days resulted in an upregulation of COX-2 mRNA. We used 5-FU doses of 0.1 *μ*g/ml (0.76 *μ*M) and 0.5 *μ*g/ml (3.8 *μ*M) for the SUM-149 cells, and 0.5 *μ*g/ml (3.8 *μ*M) and 2.5 *μ*g/ml (19.2 *μ*M) for the MDA-MB-231 cells treated over a 24 h period and observed a pronounced increase of COX-2 after 96 h hypoxia. Our results with the SUM-149 cells highlight how a combination of hypoxia and 5-FU treatment in tumors can increase COX-2 expression and its associated risks of increased invasion and metastasis^[21,37]^.

It is possible that 5-FU mediated reduction of cell viability, and the molecular changes in COX-2 are independent outcomes. Indeed COX-2 downregulation with celecoxib in a high COX-2 expressing brain metastatic variant of MDA-MB-231 cells did little to alter resistance to 5-FU^[38]^. COX-2 may not play a role in the viability response but in itself may have a multitude of phenotypic effects. Cells that survive hypoxia and 5-FU treatment may exhibit a more aggressive phenotype through the formation of HIF and COX-2. This becomes increasingly relevant in the neoadjuvant setting. In the neoadjuvant setting, celecoxib has been found to increase the chemotherapy response rate by 20%^[39]^. In a Phase II study, a combination of celecoxib and capecitabine increased time to progression and overall survival in patients with high COX-2 expressing metastatic breast cancers^[40]^. Our data support further investigation of the effects of chemotherapy and hypoxia on COX-2 expression, and the role of COX-2 in the subsequent phenotypic characteristics of these cells such as invasion and metastasis. Our data also support investigating the role of HIF-2*α* in modifying cell survival following hypoxia or therapy.
